# Evidence That Erythropoietin Modulates Neuroinflammation through Differential Action on Neurons, Astrocytes, and Microglia

**DOI:** 10.3389/fimmu.2014.00523

**Published:** 2014-10-22

**Authors:** Wesley S. Bond, Tonia S. Rex

**Affiliations:** ^1^Vanderbilt Eye Institute, Vanderbilt University Medical Center, Nashville, TN, USA; ^2^Vanderbilt Brain Institute, Vanderbilt University Medical Center, Nashville, TN, USA

**Keywords:** erythropoietin, neuroinflammation, microglia, astrocytes, signaling pathways

## Abstract

Neuroinflammation is a normal and healthy response to neuronal damage. However, excessive or chronic neuroinflammation exacerbates neurodegeneration after trauma and in progressive diseases such as Alzheimer’s, Parkinson’s, age-related macular degeneration, and glaucoma. Therefore, molecules that modulate neuroinflammation are candidates as neuroprotective agents. Erythropoietin (EPO) is a known neuroprotective agent that indirectly attenuates neuroinflammation, in part, by inhibiting neuronal apoptosis. In this review, we provide evidence that EPO also modulates neuroinflammation upstream of apoptosis by acting directly on glia. Further, the signaling induced by EPO may differ depending on cell type and context possibly as a result of activation of different receptors. While significant progress has been made in our understanding of EPO signaling, this review also identifies areas for future study in terms of the role of EPO in modulating neuroinflammation.

## Introduction

Inflammation is a physiologic response to injury and infection and is necessary for tissue healing. A similar process occurs in the central nervous system (CNS) in response to injury or disease and is termed neuroinflammation. In acute neuroinflammation, microglial cells become reactive, they phagocytose dying cells and release pro-inflammatory cytokines and chemokines to limit the area of injury [for review see Ref. (1)]. However, when neuroinflammation is severe or chronic, it can produce deleterious effects involving pro-inflammatory signaling pathways, increased oxidative stress, and death of nearby neurons. Neuroinflammation is a common mechanism influencing the severity and progression of neurodegenerative disease and injury and is, therefore, a potential target for neuroprotective therapies [for review see Ref. (2)].

**Figure 1:**
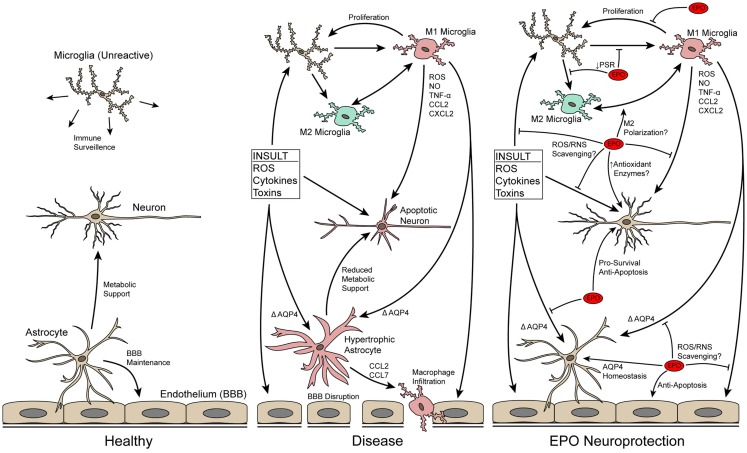
**Schematic of neuron–glia interactions in healthy and diseased CNS tissue and how EPO may protect neurons by modulating neuroinflammation**. In the normal CNS, microglia and astrocytes serve important support roles. In disease/trauma, the BBB breaks-down due to endothelial cell death and astrocyte hypertrophy, immune cells infiltrate into the CNS, microglia increase in number and become reactive, and neurons undergo apoptosis. EPO directly blocks apoptosis of neurons and preserves the BBB by blocking apoptosis of endothelial cells and decreasing astrocyte hypertrophy, thus, decreasing infiltration of immune cells. In addition, EPO may directly scavenge ROS/RNS to reduce local oxidative stress. EPO also has a direct effect on microglia, affecting their proliferative capability and possibly influencing their M1/M2 reactive state.

Erythropoietin (EPO) was originally identified as a cytokine responsible for production of red blood cells by blocking apoptosis of progenitor cells [for reviews see Ref. (3–6)]. EPO is also produced at low levels in CNS tissue, and the EPO receptor (EpoR) homodimer is expressed on most CNS cell types, including neurons, astrocytes, and microglia [for review see Ref. (7)]. In the last 20 years, EPO has proven to be effective in preventing neuronal apoptosis in a wide-range of neurodegenerative conditions in the brain, retina, and spinal cord including acute, chronic, inherited, and induced degenerations. Briefly, EPO affects the regulators of apoptosis Bax, Bad, and Bcl-2/Bcl-xL by inhibiting formation of the Bax/Bcl complex and reducing activation of effector caspases. Comprehensive reviews on the anti-apoptotic effect of EPO in the CNS are available (4, 8, 9). EPO also blocks apoptosis in retinal neurons (10–23), showing that EPO acts similarly in all CNS tissue. Since the anti-apoptotic role of EPO is well-characterized it is not the focus of this review except to note that it is well accepted that EPO decreases neuroinflammation and its damaging effects in part by blocking apoptosis [Ref. (22, 23); Figure 1]. This review will discuss recent evidence that points to additional, apoptosis-independent, actions of EPO in modulating neuroinflammation including blocking reactive oxygen/nitrogen species (ROS/RNS) and glial reactivity. Accruing evidence that the signal transduction cascades activated by EPO may differ based on cell type will also be presented.

## EPO Limits Neuroinflammation and Cell Death by Decreasing ROS/RNS Levels

Oxidative/nitrosative stress refers to the undesirable modification of proteins, lipids, and DNA mainly thought to arise from mitochondrial dysfunction [for review see Ref. (24)]. Peroxynitrite, a by-product of superoxide and nitric oxide, can cause DNA damage and ultimately lead to necrosis, which in turn drives an inflammatory response that includes microglial reactivity. Reactive microglia are particularly effective at producing and releasing ROS/RNS [for review see Ref. (25)]. Oxidative/nitrosative stress and neuroinflammation have been implicated in a myriad of disease processes and has been shown to contribute to neuronal degeneration in Alzheimer’s, Parkinson’s, traumatic brain injury [for review see Ref. (26)], and glaucoma [for review see Ref. (27)].

Treatment with EPO decreases cellular damage caused by ROS/RNS, including lipid peroxidation (28–30), protein carbonylation (30), and protein nitrosylation (31) and significant progress has been made in elucidating how this is accomplished. EPO preserves mitochondrial membrane integrity in a β-amyloid model of Alzheimer’s disease (32). It also increases levels of antioxidant enzymes by increasing levels and/or translocation of nuclear factor erythroid 2-related factor 2 (Nrf-2) to the nucleus where it binds and activates the antioxidant response element (33–36). In neurons, the increased nuclear translocation of Nrf-2 appears to be mediated by PI-3K, ERK, and JNK, but not p38/MAPK (36). Some of the antioxidant enzymes increased by EPO in terms of both levels and activity include heme oxygenase (HO-1) (33, 36), peroxiredoxin (37), glutathione peroxidase, NAD(P)H:quinone oxidoreductase 1 (NQO1) (33–35), glutamate cysteine ligase, and glutathione S-transferase (33, 35). EPO also causes increases in the *in vivo* activity of the antioxidant proteins catalase (29), superoxide dismutase (30), and glutathione peroxidase (38). However, EPO’s positive effect on levels and activity of these enzymes is not consistently observed (39, 40). For example, EPO has no effect on expression of induced nitric oxide synthase (iNOS) in cultured activated microglia (41), even though it reduces total retinal levels of iNOS in a glaucoma model (42). These data suggest that EPO’s effect on antioxidant enzyme activity may contextual or cell-specific.

Two alternative methods for reduction of oxidative/nitrosative stress by EPO have been reported. First, there is emerging biochemical evidence that EPO is capable of directly scavenging ROS/RNS (43), including a study showing that EPO protects paraquat-treated astrocytes in a superoxide dismutase knockout mouse (44). Second, in some disease processes where iron accumulation is thought to be a key mediator of oxidative damage and degeneration, such as Parkinson’s disease [for review see Ref. (45)], EPO may indirectly promote an antioxidant effect through its increase in erythrocyte production and corresponding depletion of systemic iron. This is supported by the long-observed phenomenon of EPO treatment leading to systemic iron depletion [for review see Ref. (46)]. Therefore, EPO appears to act in a multi-pronged way to mitigate ROS/RNS levels thus preventing downstream damaging effects on cells that lead to apoptosis (Figure 1).

## EPO Decreases Recruitment and Infiltration of Immune Cells

In neurodegenerative conditions, immune cells are recruited to the area of injury by the release of chemokines from the damaged neuronal tissue [for review see Ref. (47)]. Expression of two of these chemokines, CXCL2 and CCL7, is decreased by treatment with EPO in a stroke model (48). This suggests that EPO may limit recruitment of immune cells, which would in turn decrease release of pro-inflammatory cytokines into the CNS and migration of immune cells into the tissue. Migration of immune cells into the CNS occurs as a result of blood–brain/retina barrier (BBB/BRB) disruption after CNS trauma or in neurodegenerative diseases due to microvascular endothelial cell death (49), tight junction structural changes [for review see Ref. (50)], and astrocyte hypertrophy [Figure 1; for reviews see Ref. (51, 52)]. EPO preserves the BBB/BRB in multiple models (21, 53, 54) by blocking apoptosis of microvascular endothelial cells [for review see Ref. (9)] and astrocyte hypertrophy. Here, we will focus on the less well-characterized role of EPO in blocking astrocyte hypertrophy.

Dysregulation of aquaporin-4 is implicated in astrocyte swelling and disruption of the BBB/BRB in knockout models (55), though this effect is not consistently observed (56, 57). Astrocytes respond to EPO by activating JNK and p38-MAPK (53, 58), leading to modulation of aquaporin-4 levels (53, 58–60), decreased glial swelling (58), and reduced BBB permeability (54, 59). EPO also increases levels of tight junction proteins in these cells via activation of the MAPK cascade (53). Upregulation of the stress-induced intermediate filament protein, GFAP, is associated with glial hypertrophy and is also decreased by treatment with EPO (19, 60, 61). Interestingly, modulation of GFAP levels by EPO is independent of the MAPK pathway (60). Others have reported that EPO neither activates JAK2, Akt, ERK nor STAT in cultured astrocytes (53, 62), suggesting that these signaling molecules also might not be involved.

## EPO Decreases Microglial Proliferation and Reactivity

Microglia are the key mediators of neuroinflammation. In the injured/degenerative CNS, innate microglia proliferate and convert into the M1 (pro-inflammatory) and M2 (alternative) reactive states in a manner similar to systemic macrophages [for review see Ref. (63)]. Reactive microglia can mediate both pro-inflammatory and anti-inflammatory states depending on their particular reactive state and the corresponding milieu of cytokines and chemokines released. The role of these cells is complex and current research suggests that while an overactive microglia response is deleterious, blocking it entirely can also be detrimental [for review see Ref. (64)]. In this review, we will focus on the damaging effects of chronically reactive microglia.

Erythropoietin may directly influence the reactive state of the CNS microglia (Figure 1). One of the distinguishing features of a reactive microglia is its ability to phagocytose dying neurons. Apoptotic neurons increase levels of phosphatidylserine on the outer leaflet of the plasma membrane. Recognition of these residues is a key step in microglial phagocytosis. Reactive microglia express higher levels of the phosphatidylserine receptor [PSR; (65)]. EPO treatment decreases levels of the PSR on the microglial plasma membrane *in vitro* (66). This suggests that EPO treatment decreases the ability of microglial cells to phagocytose dying neurons. Active phagocytosis of apoptotic cells by microglia suppresses production of pro-inflammatory cytokines (65). Therefore, the decrease in PSR could suggest that EPO induces a pro-inflammatory state in the CNS. However, treatment with EPO leads to lower levels of pro-inflammatory cytokines in *in vivo* studies of neurodegenerative conditions [Ref. (22, 67–69); Figure 1]. The decrease in pro-inflammatory cytokines by EPO is not due to a direct block in production based on *in vitro* experiments (22, 41, 70). This suggests that the lower levels detected in the *in vivo* studies is likely an indirectly consequence of EPO limiting the number of reactive microglia present. In fact, fewer proliferating cellular nuclear antigen-positive primary microglial cells are detected after treatment with EPO (66). In summary, the data suggest that EPO redirects microglia back to or maintains microglia in a normal state and prevents microglial proliferation.

## EPO Induces Signaling in Microglia and Macrophages

Microglia share similar characteristics with systemic macrophages and both can be found in the injured/degenerative CNS. Macrophages can infiltrate into the CNS where they take on a reactive microglial-like morphology. Like microglial cells, macrophages secrete neuroinflammatory cytokines and phagocytose dying cells. Therefore, they play an important role in chronic neuroinflammation. We will compare and contrast signaling by EPO in microglia and macrophages.

**Figure 2:**
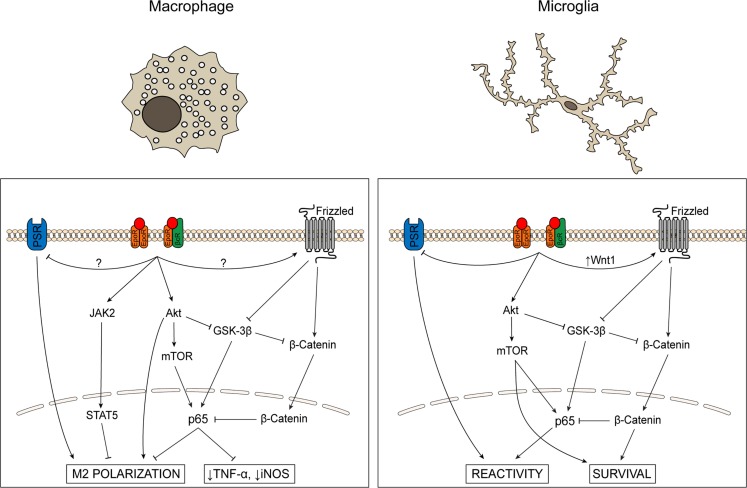
**Schematic comparing signaling cascades activated by EPO in microglia and macrophages**. The role of EPO in macrophages and microglia has primarily been evaluated in the context of inflammatory state and cell survival, respectively. In macrophages, EPO activates JAK/STAT and Akt signaling, inhibits GSK-3β activity, modulates NF-κB p65, decreases levels of pro-inflammatory cytokines and iNOS, and promotes phagocytosis and M2 activation state polarization. In microglia, EPO promotes cell survival, inhibits GSK-3β activity, and increases NF-κB p65 via PI-3K/Akt while simultaneously activating the Wnt-1/β-catenin pathway that results in sequestration of NF-κB in the nucleus. EPO also decreases PSR levels, suggesting decreased phagocytosis.

The EpoR is expressed on both macrophages and microglia (71–74). The investigations performed, to date, on microglial cells in culture have focused on the ability of EPO to block cell death rather than an effect on altering the reactive state of these cells. While the physiological relevance of microglial cell survival as opposed to proliferation or reactivity is unclear, these studies at a minimum demonstrate that EPO can activate signaling cascades in microglia. EPO-mediated protection of EOC-2 microglia-derived cells requires Wnt-1, PI-3K, and Akt, and also involves mTOR, and p70S6K [Ref. (71–73); Figure 2]. As a result of activation of the PI-3K/Akt pathway, the p65 subunit of NF-κB is translocated to the nucleus (73). Surprisingly, there is a concomitant increase in levels of Wnt-1 and de-activation of glycogen synthase kinase 3 (GSK-3α/β) by phosphorylation, which results in activation (phosphorylation) of β-catenin. Activated β-catenin translocates to the nucleus where it can sequester NF-κB (75), preventing it from activating gene expression (Figure 2). It is unclear why EPO would seemingly activate two conflicting pathways. One possibility is that EPO modulates each pathway in a context-dependent manner to modulate neuroinflammation, either promoting a neuroinflammatory state (activation of NF-κB) or decreasing it (sequestration of NF-κB by β-catenin). Additional studies to assess signaling pathways induced by EPO in primary microglia, particularly in the context of microglial proliferation and polarization are warranted.

In contrast, the EPO studies on cultured macrophages assess the activation state rather than survival of these cells. As in microglial cells, treatment with EPO activates the Akt/mTOR/NF-κB pathway. This pathway is implicated in shifting macrophage activation state polarization from M1 to M2 (76). For example, treatment of cultured macrophages with an EPO peptide caused a dose-dependent increase in phagocytosis and a corresponding decrease in TNF-α, suggesting that EPO shifts these cells from a pro-inflammatory to a phagocytic state but does not return them to a non-reactive state (77). Also, unlike in microglial cells, EPO inhibits NF-κB p65 in macrophages, leading to lower levels of TNF-α and NO (78). Further studies are needed to understand how EPO decreases NF-κB activity in macrophages while increasing it in microglia. It is feasible that the same signaling balance between the PI-3K/Akt and Wnt/β-catenin pathways are present in both microglial cells and macrophages, but that the balance is shifted to the Wnt/β-catenin pathway in activated macrophages, resulting in sequestration/inhibition of NF-κB. However, while activation of β-catenin by Frizzled has been demonstrated in macrophages (79), to date, no studies have investigated if EPO affects this pathway or if EPO has any effect on Wnt-1. Given the phenotypic similarity between systemic macrophages and resident microglia, as well as the identification of common signaling pathways affected by EPO in both cell types, the potential role of EPO in modulating additional pathways in microglia that have been identified in macrophages should be investigated.

## EPO Signaling may be Cell Type and Context Dependent

The molecular pathways activated by EPO for neuroprotection are an area of active investigation. Many studies have reported changes in a myriad of signaling pathways in complex neuronal tissue *in situ* after a variety of insults and treatment with EPO (10, 21, 80–87). Since the EpoR is expressed in glia and endothelial cells in addition to neurons [for review see Ref. (7)], studies in primary cell cultures are helpful to parse out the role of EPO in these different cell types. These studies, when compared, suggest that EPO may activate different signal transduction cascades depending on cell type. For example, results from a combination of studies suggest that the p38-MAPK pathway is activated by EPO in astrocytes, but not in neurons. Whole tissue studies detected activation of p38-MAPK by EPO and, as mentioned above, EPO preserves astrocyte function at the BBB by activation of this pathway (53, 58). However, blocking activation of the p38-MAPK pathway *in vivo* had no effect on neuronal survival (87). Taken together, these observations suggest that functional effects of EPO via activation of p38-MAPK occur primarily in non-neuronal cells, including astrocytes.

Disparate signaling pathways may be initiated by EPO as a result of binding to different receptors. In hematopoietic cells, EPO activates the EpoR homodimer to induce downstream signaling and block apoptosis (88–92). In non-hematopoietic tissue, EPO may enact neuroprotection via an EpoR, interleukin beta common receptor (βcR) heterodimer (93). This is supported by evidence that the EpoR can associate with the βcR (94), and that binding of the EpoR to a dimerization-dependent, constitutively active βcR mutant initiates downstream signaling cascades (95). However, expression of the EpoR and the βcR does not appear to significantly overlap in the brain, despite an increase in microglial βcR expression following injury (96). This could imply that while a few cells or specific cell types may respond to EPO via an EpoR, βcR complex, the majority of cells may not. In fact, EPO could potentially act on an additional as of yet unidentified receptor. Neuronal survival was recently achieved by EPO therapy in mice that lack neuronal EpoR expression (97), thus suggesting that the EpoR is not necessary for blocking neuronal apoptosis. Further, forms of EPO that neither bind the EpoR nor initiate erythropoiesis are still neuroprotective (15, 98–101). Understanding whether EPO acts through canonical or non-canonical receptors, or independently of receptor interaction (i.e., direct ROS/RNS scavenging) is essential for developing new target-directed therapies and therefore should continue to be an area of active investigation.

In addition to direct binding of EPO to different receptors, activation of the EpoR can activate other surface receptors and channels. Signal transduction pathways downstream of EPO are significantly modulated by cross-talk with other surface receptors and cytosolic proteins in erythroid progenitors [for review see Ref. (5)]. We have already discussed the potential influence of cross-talk with the Wnt/β-catenin pathway in influencing downstream effects of EPO in microglial cells. Another example is the calcium channel, TRPC2 (102, 103), which facilitates calmodulin-dependent enhancement of EpoR-associated JAK2 signaling in erythroid progenitor cells (104). TRPC2 channels are also expressed in the CNS and expression may vary between cell types, which could also contribute to cell type specific responses to EPO (105, 106). This observation supports cell type specific signal transduction events downstream of even canonical EpoR signaling, dependent on cell type specific expression of these other proteins. Altogether these data suggest that EPO may be able to activate different receptors and that even signaling through the canonical EpoR homodimer may differ depending on cell type. Additional studies are needed to determine if these co-regulators of EPO signaling vary in expression among different CNS cell types and whether these factors play a role in differing responses to EPO.

## Conclusion

Erythropoietin is a pleiotropic protein, it influences erythropoiesis, BBB/BRB health, ROS/RNS levels, apoptosis, and glial reactivity seemingly simultaneously. EPO blocks apoptosis in a wide-range of cell types, including neurons, and it appears to be effective in a wide-range of neurodegenerative conditions. By preventing apoptosis, EPO indirectly decreases chronic neuroinflammation. In this review, we show evidence that EPO also modulates neuroinflammation by decreasing levels of ROS/RNS, limiting microglial infiltration by preserving the health of the microvascular endothelial cells and astrocytes at the BBB/BRB, and by acting directly on microglial cells to block proliferation and influence their reactive state. We propose that EPO may activate different signal transduction cascades in a context-dependent, cell type specific manner to enact its diverse functions. A caveat of this analysis is that not all signaling molecules have been assessed in all cell types. For example, Wnt-1 activation by EPO was reported in vascular endothelial cells (107) and in cultured microglia (71), but has not been assessed in astrocyte or neuronal cultures. There has been amazing progress in this field in the last 10 years, but there is still much to be understood about this complex, pleiotropic cytokine. Understanding the mechanism by which EPO modulates neuroinflammation may lead to novel therapeutic strategies for the treatment of neurodegenerative diseases and injuries.

## Conflict of Interest Statement

The authors declare that the research was conducted in the absence of any commercial or financial relationships that could be construed as a potential conflict of interest.
